# Optimizing Text Messages to Promote Engagement With Internet Smoking Cessation Treatment: Results From a Factorial Screening Experiment

**DOI:** 10.2196/17734

**Published:** 2020-04-02

**Authors:** Amanda L Graham, George D Papandonatos, Megan A Jacobs, Michael S Amato, Sarah Cha, Amy M Cohn, Lorien C Abroms, Robyn Whittaker

**Affiliations:** 1 Innovations Center Truth Initiative Washington, DC United States; 2 Mayo Clinic College of Medicine and Science Rochester, MN United States; 3 Center for Statistical Sciences Brown University Providence, RI United States; 4 Oklahoma Tobacco Research Center University of Oklahoma Health Sciences Center Oklahoma City, OK United States; 5 Department of Prevention and Community Health Milken Institute School of Public Health The George Washington University Washington, DC United States; 6 National Institute for Health Innovation University of Auckland Auckland New Zealand

**Keywords:** smoking cessation, tobacco dependence, internet, text messaging

## Abstract

**Background:**

Smoking remains a leading cause of preventable death and illness. Internet interventions for smoking cessation have the potential to significantly impact public health, given their broad reach and proven effectiveness. Given the dose-response association between engagement and behavior change, identifying strategies to promote engagement is a priority across digital health interventions. Text messaging is a proven smoking cessation treatment modality and a powerful strategy to increase intervention engagement in other areas of health, but it has not been tested as an engagement strategy for a digital cessation intervention.

**Objective:**

This study examined the impact of 4 experimental text message design factors on adult smokers’ engagement with an internet smoking cessation program.

**Methods:**

We conducted a 2×2×2×2 full factorial screening experiment wherein 864 participants were randomized to 1 of 16 experimental conditions after registering with a free internet smoking cessation program and enrolling in its automated text message program. Experimental factors were *personalization* (on/off), *integration* between the web and text message platforms (on/off), *dynamic tailoring* of intervention content based on user engagement (on/off), and *message intensity* (tapered vs abrupt drop-off). Primary outcomes were 3-month measures of engagement (ie, page views, time on site, and return visits to the website) as well as use of 6 interactive features of the internet program. All metrics were automatically tracked; there were no missing data.

**Results:**

Main effects were detected for *integration* and *dynamic tailoring*. *Integration* significantly increased interactive feature use by participants, whereas *dynamic tailoring* increased the number of features used and page views. No main effects were found for *message intensity* or *personalization* alone, although several synergistic interactions with other experimental features were observed. Synergistic effects, when all experimental factors were active, resulted in the highest rates of interactive feature use and the greatest proportion of participants at high levels of engagement. Measured in terms of standardized mean differences (SMDs), effects on interactive feature use were highest for Build Support System (SMD 0.56; 95% CI 0.27 to 0.81), Choose Quit Smoking Aid (SMD 0.38; 95% CI 0.10 to 0.66), and Track Smoking Triggers (SMD 0.33; 95% CI 0.05 to 0.61). Among the engagement metrics, the largest effects were on overall feature utilization (SMD 0.33; 95% CI 0.06 to 0.59) and time on site (SMD 0.29; 95% CI 0.01 to 0.57). As no SMD >0.30 was observed for main effects on any outcome, results suggest that for some outcomes, the combined intervention was stronger than individual factors alone.

**Conclusions:**

This factorial experiment demonstrates the effectiveness of text messaging as a strategy to increase engagement with an internet smoking cessation intervention, resulting in greater overall intervention dose and greater exposure to the core components of tobacco dependence treatment that can promote abstinence.

**Trial Registration:**

ClinicalTrials.gov NCT02585206; https://clinicaltrials.gov/ct2/show/NCT02585206.

**International Registered Report Identifier (IRRID):**

RR2-10.1136/bmjopen-2015-010687

## Introduction

### Background

Internet-based interventions for smoking cessation have the potential to significantly impact public health, given their broad reach and proven effectiveness. Nine of 10 adults in the United States have internet access [1], more than one-third of all smokers—12.4 million individuals—look online each year to quit smoking [2], and hundreds of thousands enroll in freely available programs [3,4]. Systematic reviews and meta-analyses have demonstrated the effectiveness of tailored and interactive internet interventions for smoking cessation [5]. However, a sizable proportion of smokers disengage early from internet programs without being exposed to the content or features that can promote abstinence. Indeed, low levels of engagement with internet interventions have been documented across a range of health behaviors [6]. Given the evidence of a dose-response association between engagement and behavior change outcomes [7-9], identifying strategies to promote engagement has been noted as a priority across digital health interventions [10-12].

The relationship of engagement to outcomes is complex and includes both behavioral and cognitive dimensions [11,13]. Engagement with an internet intervention can be usefully conceptualized into 3 phases [14], although users’ progress through the phases is often nonlinear. In the first phase, an individual decides to visit a website to determine its relevance and potential utility. In the second phase, the individual uses a part of the intervention. In the third phase, the individual returns to engage more fully with the intervention. This study aimed to influence engagement at this third phase by using prompts and reminders delivered via text messages. Text messaging is a proven intervention modality to promote smoking cessation [15] and a powerful strategy to increase intervention engagement [16,17]. Across most demographic groups, a majority of individuals own a mobile phone and use text messaging [18], including economically disadvantaged groups among whom tobacco use is more prevalent. However, to date, text messaging has not been tested specifically as an engagement strategy for internet cessation interventions [10], and little is known about how best to design such text messages [19].

The Multiphase Optimization Strategy (MOST) is a method for systematically building and evaluating interventions to ensure they comprise active components delivered in optimal doses [20]. The screening phase of MOST is designed to determine which intervention components are active (ie, make a difference in the target outcome) and should be retained, and which are inactive and should be discarded. This is accomplished efficiently through a randomized experiment involving a factorial design, which allows for the examination of several design factors simultaneously. As they are scalable and can automate the delivery of many experimental conditions, internet interventions are well suited to conduct such experiments [21]. Guided by the principles of MOST, we conducted a screening experiment to evaluate 4 experimental factors hypothesized to promote engagement with an internet smoking cessation program.

*Personalization* incorporates user-specific elements (eg, name) to enhance the personal relevance of messaging. People are more likely to actively process information if they perceive it to be personally relevant [22]. Personalization can increase smokers’ attention to written information and the perceived quality of that information [23,24] and is a desired and expected feature of text messaging [25]. Supported by prior literature [26], we hypothesized that text messages that incorporate personalized content would be more efficacious than generic ones.

*Integration* refers to the ability to interact with an intervention platform through the web and text messages, accomplished by sharing data between systems. This approach leverages the unique and combined advantages of these 2 different modalities to create a seamless user experience. Internet interventions can be used to deliver multimedia content but require users to initiate contact, whereas text messages are a powerful form of push notification that have a 98% open rate, with 90% of messages being read within 3 min [27]. A 2010 meta-analysis found that the effectiveness of internet interventions for a variety of health behaviors was enhanced by adding text messaging [28], but these early studies and others [29] most often delivered text messaging in parallel to a web-based intervention with little integration between the 2 modalities. We hypothesized that an intervention that allows smokers to interact with the tools and content of an internet program via text messages would be more effective in promoting treatment engagement than delivering text messages in parallel to an internet intervention.

*Dynamic Tailoring* delivers individually tailored feedback that adapts over time to a smoker’s needs. Research over several decades supports the superiority of individually tailored messaging over generic, one-size-fits-all messaging in improving behavior change outcomes [26] and in promoting intervention engagement [30]. However, it typically involves messaging around static, theory-driven psychosocial constructs (eg, readiness to quit and self-efficacy) gathered at the outset of an intervention. Few studies have dynamically tailored communications to deliver feedback based on a user’s pattern of intervention engagement [31]. We hypothesized that messages tailored to a user’s pattern of engagement to encourage the exploration of components they have not yet used and prompt continued engagement with the ones they have would yield higher engagement than messages without this kind of feedback.

*Message intensity* refers to the delivery schedule of text messages. One of the largest randomized smoking cessation trials demonstrating the effectiveness of a text message intervention [32] delivered 5 messages a day for the first 5 weeks, followed by an abrupt drop to just 3 messages per week for the next 26 weeks. However, a meta-analysis of health behavior change interventions found that the largest effect size was observed for text message interventions with tapered intensity (ie, gradually decreasing content delivery [26]). We set tapered intensity as the active form of this factor and hypothesized that it would make message delivery more salient and impactful than a fixed schedule of messages followed by an abrupt drop-off.

### Objective

To summarize, this factorial screening experiment evaluated the impact of *personalization*, *integration*, *dynamic tailoring*, and *message intensity* on engagement with an internet smoking cessation program. We hypothesized that the active form of each experimental factor would yield higher rates of engagement with one or more features of the program and overall metrics of engagement.

## Methods

### Experimental Design

This full factorial experiment had 4 factors, each of which was implemented at 2 levels: *personalization* (on/off), *integration* (on/off), *dynamic tailoring* (on/off), and *message intensity* (tapered/abrupt). The factors were designed to be compatible with each other but also to deliver a coherent intervention when implemented singularly. All participants had full access to the website to use as they desired. In addition, they were randomized by a computer algorithm to one of the 2^4^=16 experimental conditions, stratified by whether they enrolled on a desktop/mobile because mobile access to the website may influence engagement. The use of the website and text messages was automatically tracked for 3 months to allow sufficient time to examine the impact of the text message intervention on intervention engagement because most nonusage attrition happens within this period [33]. There was no involvement by research staff, and there were no missing data.

### Procedure

This fully automated experiment was conducted within BecomeAnEX (*EX*), a free, evidence-based smoking cessation program developed in 2008 by Truth Initiative in collaboration with Mayo Clinic. Since it launched, more than 800,000 tobacco users have registered on the site. Approximately 80% of newly registered users sign up for text messaging. As described previously [34], newly registered users who met study eligibility criteria were randomized to 1 of the 16 experimental arms. Eligibility criteria were current smoking (every day/some days), US residence, aged 18 years or older, and enrollment in the EX text message program during website registration. To register on EX, users must agree to the terms of use and privacy policy, which state that (1) Truth Initiative automatically collects information about use of the site, (2) information is used for research and quality improvement purposes, and (3) personal information is kept confidential. To enroll in the EX text message program, users enter their mobile number and explicitly consent to receive text messages during website registration. The study was conducted as a quality improvement project, meaning that eligible individuals were automatically randomized; no recruitment information was presented, and no study informed consent was solicited. The Chesapeake institutional review board approved the trial protocol (CR00086431).

### Web-Based Cessation Program

EX, which is accessible on any web-enabled device, was designed around tobacco dependence treatment guidelines [35], Social Cognitive Theory [36], and the Mayo Clinic model for engaging tobacco users in cessation treatment [37]. At the time of this study, users could engage with 6 interactive features: (1) Set Quit Date assists users in selecting a quit date, (2) Track Smoking Triggers allows users to track cigarettes and identify personal smoking triggers associated with smoking, (3) Beat Smoking Triggers encourages identification of strategies to dissociate cigarettes from triggers, (4) Choose Quit Smoking Aid educates users about medication and helps them create a medication plan, (5) Build Support System discusses the importance of social support and encourages users to identify supportive friends/family, and (6) EX Community introduces users to a large online social network of current and former smokers. Higher levels of engagement with the site and its features are associated with greater odds of quitting [8,38].

### Text Messaging Intervention

The standard EX text message program is a 90-day program that is fully automated. Also designed around tobacco cessation treatment guidelines [35] and Social Cognitive Theory [36], like the EX website, the program is designed to enhance motivation for quitting, educate users about addiction, encourage the use of evidence-based cessation methods (eg, nicotine replacement therapy and peer support), prepare them for quitting by building confidence and coping skills, praise success, reinforce the benefits of quitting, and support users in maintaining abstinence. The program is interactive, with bidirectional messages (eg, true/false, multiple-choice, and yes/no questions) and keywords that allow users to request on-demand support (eg, CRAVE, SLIP, and MOOD), additional detail about specific topics (MORE), or to unenroll (STOP). Approximately 10% of messages contain URLs to the EX website for additional information. The program is tailored to a participant’s quit date, should they choose to set one. Users can set and reset their quit date as often as they like either via the website or text messages. This basic level of integration is expected by users and was common across all experimental conditions.

Text messages focused on promoting engagement were added to this standard program according to the experimental factors described in the following section. These messages were designed to encourage or facilitate the use of the interactive features of EX and promote sustained engagement.

### Experimental Factors

#### Personalization

Half of the participants received text messages personalized with their EX username, chosen during registration on the website. The remaining participants received generic messages with no personalization.

#### Integration

Participants with this experimental factor turned *on* were able to engage with interactive features of EX through special keywords and interactive messages. For example, the use of Track Smoking Triggers and Beat Smoking Triggers was facilitated as follows:

System: Quitting is a journey—not an event. If you’ve had a slip, learn about your triggers by texting back SMOKED or going to http://bit.ly/trackcig and noting your high-risk situations.

User: SMOKED.

System: Ok, you smoked—we saved it in your EX Plan. Reply with the trigger that made you want to smoke.

User: Sitting in traffic.

System: Got it, we’ve noted it in your EX Plan. To make a plan to separate from this trigger, text back COPE.

User: COPE.

System: You said that Sitting in traffic was a trigger for you. What else can you do instead of smoke the next time that trigger comes up? Reply with your plan, the more detail the better!

User: Listen to music.

System: Great idea! It’s now in your EX Plan at http://bit.ly/beattrigger so you can always reference it if that trigger pops up.

As seen in this exchange, interaction with the platform via text messages was reflected on the website at the user’s next visit. This approach was used for the first 5 interactive features described earlier. To facilitate engagement with the EX Community via text message, a slightly different approach was required for technical reasons. Once a participant had accessed the community via the website, a special keyword (TIPS) allowed the user to request advice and guidance from community members that had been manually curated from actual community posts. For participants with the integration feature turned off, there was no integration of the website and text message programs beyond the quit date feature, and special keywords were not available.

#### Dynamic Tailoring

Half of the participants received messages tailored to real-time engagement data from EX. Messages reinforced actions that they had already taken or prompted the exploration of features they had not yet used. The remaining participants received standard messages that were agnostic to prior use of EX.

#### Message Intensity

The intervention duration was 12 weeks for both groups. Both groups received 2 messages per day for the first 3 days of the program to ensure a standardized onboarding experience, and in both program versions, approximately half of all engagement messages solicited a response from users. Participants randomized to *tapered* intensity received a total of 69 engagement messages delivered as follows: weeks 1 to 2, 14 messages per week; weeks 3 to 4, 7 messages per week; weeks 5 to 8, 4 messages per week; weeks 9 to 11, 3 messages per week; and week 12, 2 messages. Participants randomized to an *abrupt* intensity drop-off received a total of 28 engagement messages, which were delivered as follows: week 1, 8 messages; week 2, 4 messages; weeks 3 to 8, 2 messages per week; weeks 9 to 12, 1 message per week.

### Sources of Data

Gender, age, and smoking status (*every day* or *some days*) entered during website registration were extracted from the EX database. Website utilization metrics were extracted at 3-month postrandomization and included measures of website engagement (number of website visits, time on site in minutes, and page views) and the use of the 6 interactive features described earlier. Text message data were also extracted at 3-month postrandomization and included the number of messages received and sent by participants, the use of the 6 interactive features described earlier via text messages, the use of keywords, and the date of unenrollment.

### Analytic Plan

A full factorial design was used in the study design phase [34]. The primary outcome used for sample size calculations was a composite engagement score, with weights given by the regression coefficients of a logistic regression model developed to measure the effects of website engagement on 3-month abstinence rates in the control arm of a previous randomized trial by our group [7]. This composite engagement score had the advantage of being continuously distributed, even if some of the individual engagement metrics were binary or count data. A priori sample size calculations based on a normal approximation to the distribution of the composite score determined that a sample of 864 (n=432 per factor level) participants would allow us to detect small main effects (*d*=0.25) or moderate second-order interactions (*d*=0.50) on normalizing transformations of this composite outcome. Power was set at 80% at a 2-sided significance level of alpha of .05 out of 10 (multiplicity adjustment based on 4 main effects and 6 two-way interactions in a factorial model, with third- and fourth-order interactions excluded a priori).

In carrying out our original analytic plan [34], we made 3 post hoc modifications. First, ongoing enhancements to the EX website led us to question the applicability of the weights of the original composite engagement metric. We decided to analyze our engagement metrics separately and to identify common patterns in standardized factorial effects across the full set of engagement metrics. Our primary outcomes were (1) number of interactive features used through the web or text messages, (2) total time spent on the website, (3) number of page views, and (4) return visits to the website (ie, postregistration). This approach is consistent with the multidimensional nature of engagement and numerous systematic reviews and meta-analyses that have called for more standardized engagement metrics to advance the field [10-12,30,39,40]. Our intent was to ensure that study effect sizes could be included in pooled analyses.

Second, evidence of synergistic interactions led us to supplement analyses focused on individual experimental factors with between-arm comparisons that capture the joint effect of multiple terms in the full factorial model, with the hope of identifying an arm with superior performance across all engagement metrics. If such an arm could be identified, the need to reestimate weights for a composite metric would become moot: any arm that dominated each available metric would also dominate their weighted average. To facilitate such between-arm comparisons, we presented CIs for each engagement metric for all 16 arm-specific means, with the confidence level adjusted so that an overlap in the respective CIs can be interpreted as lack of significant pairwise differences between the arms being compared at the alpha value of .05 significance level. Unlike cases where an arm-specific mean is being compared with constant, pairwise comparisons of means based on the overlap method involve uncertainty in the centers of both CIs under inspection [41]. In such cases, 2 arms may still be significantly different from one another at an alpha value of .05, even if their 95% CIs overlap. To correctly assess the presence of significant pairwise differences based on the overlap rule, we have employed narrower intervals whose individual confidence levels were set to about 83.5% under normality [42]. CIs were first calculated in the scale of the continuously and normally distributed linear predictor and then back-transformed to the original outcome scale.

Third, large skewness observed in time on site, page views, and website visits led us to transform the data to reduce the impact of outliers in the final model. Although a logarithmic transformation would have served this purpose, it would have changed the interpretation of the main effect of each factor in the original scale to an average of arm-specific medians. We dichotomized these variables instead at cut points that bifurcated the sample at an approximately 1:2 ratio. The cut points were one or more unique website visits (333/864, 38.5% of the sample), 15 or more minutes of website use (257/864, 29.8% of the sample), and 25 or more page views (278/864, 32.2% of the sample). Additional sensitivity analyses examined the robustness of the findings to variation in these cutoffs.

Use (yes/no) of specific interactive features and overall engagement metrics were analyzed via logistic regression except for interactive feature utilization, a count variable analyzed via quasi-Poisson regression with a scale parameter, φ, to account for under- or overdispersion. CIs for marginal factor effects were estimated via a parametric percentile bootstrap procedure [43] with 1 million replicates. Effect size calculations were based on standardized mean differences (SMDs) between high and low levels of experimental factors when calculating main effects and between arms 1 and 16 when calculating the full impact of the intervention, including main effects and higher order interactions. Arm 1 was defined with all experimental factors *off*, whereas Arm 16 was defined by all experimental factors *on*. SMDs for frequency counts were calculated as (μ_1_−μ_2_)/[φ(μ_1_+μ_2_)]^1/2^, where μ_1_ and μ_2_ were the sample means of each comparison group. SMDs for binary outcomes were calculated as (p_1_−p_2_)/[p_1_×q_1_+p_2_×q_2_]^1/2^, where p_1_=1−q_1_ and p_2_=1−q_2_ were the sample outcome prevalence of each comparison group. All analyses were conducted using the *glm* function in R version 3.6.1 (R Foundation).

## Results

### Participants

Between March 29 and June 5, 2018, 864 newly registered users on EX who met the study eligibility criteria were randomized. Of those, 83.4% (721/864) enrolled on a mobile device, and 16.6% (143/864) enrolled on a desktop. Most (844/864, 97.7%) participants were every day smokers, and 2.3% (20/864) of the participants were some day smokers. The sample was predominantly female (637/864, 73.7%). Age distribution was as follows: 18 to 30 years (182/864, 21.1%), 31 to 44 years (322/864, 37.3%), 45 to 65 years (313/864, 36.2%), and 65 years and older (47/864, 5.4%). No between-arm differences were observed for any of the abovementioned variables (all values for *P*>.15).

### Intervention Engagement

Of the 864 participants randomized, 461 (53.5%) completed the full 90-day text message program. Among participants who unenrolled, the median day of unenrollment was 8 days postrandomization (IQR 3-22). On average, study participants used, on average, 2.40 (SD 1.41) of the 6 targeted interactive features. Use by feature was as follows: Set Quit Date, 85.8% (741/864); Track Smoking Triggers, 55.4% (479/864); Choose Quit Smoking Aid, 37.0% (320/864); Visit Community, 33.4% (289/864); Beat Smoking Triggers, 15.3% (132/864); and Build Support System, 12.7% (110/864). Study participants received a median of 87 text messages (IQR 27-160) during the 3-month intervention period and sent a median of 4 text messages (IQR 3-12). One-fourth (213/864, 24.7%) of the sample used one or more keywords: MOOD (76/864, 8.8%), HELP (74/864, 8.6%), CRAVE (70/864, 8.1%), SLIP (60/864, 6.9%), SOS (34/864, 3.9%). Among participants randomized to the active integration arms, 11.6% used at least one special keyword: SMOKED (61/864, 7.1%), COPE (46/864, 5.3%), TIPS (27/864, 3.1%), TRIGGER (25/864, 2.9%), MEDS (14/864, 1.6%). Participants in the active integration arms used standard keywords at similar rates to other participants (all differences <1 percentage point).

### Engagement Outcomes

Table 1 (interactive features) and Table 2 (key engagement metrics) reflect the study findings under the original analytic plan. They show average response levels at *on* and *off* levels of each experimental factor and raw mean differences that correspond to marginal factor effects. SMDs are also included, as they allow us to calibrate the clinical significance of our nominal *P* values. In this study, *P*<.001 corresponds to SMDs ranging from 0.17 to 0.28 (small effects in Cohen nomenclature [44]), whereas *P* values greater than .001 and less than .05 correspond to SMDs ranging from 0.09 to 0.13.

As seen in Table 1, *integration* is the strongest experimental factor affecting interactive feature utilization, raising usage rates of Choose Quit Smoking Aid by 18.7 percentage points (95% CI 12.5 to 24.8) and Build Support System by 11.8 percentage points (95% CI 7.2 to 16.4). Altogether, *integration* raised the average number of interactive features used by participants by 0.36 (95% CI 0.16 to 0.57) when the study-wide mean did not exceed 2.4 features (median 2, IQR 1-3).

Table 2 suggests that *dynamic tailoring* was the experimental factor with broadest impact, in that it raised both the average number of interactive features used by 0.29 (95% CI 0.09 to 0.50) and the probability of higher engagement levels by 7.3 percentage points for page views (95% CI 1.1 to 13.5). Its beneficial effect on interactive feature use appears driven by similar increases in the rates of Build Support System (6.3 points; 95% CI 1.7 to 10.9), Track Smoking Triggers (6.6 points; 95% CI 0.1 to 13.1), and Beat Smoking Triggers (5.4 points, 95% CI 0.5 to 10.3).

**Table 1:** Marginal effects of experimental design factors on interactive feature utilization rates (95% CI).

Factor		Interactive feature					
		Set Quit Date	Choose Quit Smoking Aid	Build Support System	Track Smoking Triggers	Beat Smoking Triggers	Visit Community
**Personalization**							
	On	84.5 (81.1 to 87.9)	35.2 (30.9 to 39.6)	14.6 (11.2 to 17.9)	55.0 (50.4 to 59.5)	16.8 (13.3 to 20.4)	32.5 (28.2 to 36.8)
	Off	85.7 (82.3 to 89.0)	39.3 (34.9 to 43.7)	12.3 (9.1 to 15.5)	55.7 (51.1 to 60.2)	15.0 (11.6 to 18.4)	35.0 (30.6 to 39.4)
	Raw difference	−1.1 (−5.9 to 3.6)	−4.1 (−10.3 to 2.1)	2.3 (−2.3 to 6.9)	−0.7 (−7.1 to 5.8)	1.8 (−3.1 to 6.7)	−2.5 (−8.7 to 3.7)
	SMD^a^	−0.02 (−0.12 to 0.07)	−0.06 (−0.15 to 0.03)	0.05 (−0.05 to 0.14)	−0.01 (−0.10 to 0.08)	0.04 (−0.06 to 0.13)	−0.04 (−0.13 to 0.06)
**Integration**							
	On	84.5 (81.1 to 88.0)	46.6 (42.0 to 52.2)	19.3 (15.6 to 23.0)	58.2 (53.6 to 62.7)	17.7 (14.1 to 21.3)	32.3 (28.0 to 36.6)
	Off	85.7 (82.3 to 89.0)	27.9 (23.8 to 32.1)	7.5 (4.8 to 10.3)	52.5 (47.9 to 57.1)	14.1 (10.8 to 17.4)	35.2 (30.8 to 39.6)
	Raw difference	−1.1 (−5.9 to 3.7)	18.7 (12.5 to 24.8)^b^	11.8 (7.2 to 16.4)^b^	5.7 (−0.8 to 12.1)	3.6 (−1.3 to 8.5)	−2.9 (−9.1 to 3.2)
	SMD	−0.02 (−0.12 to 0.07)	0.28 (0.18 to 0.38)^b^	0.25 (0.15 to 0.34)^b^	0.08 (−0.01 to 0.17)	0.07 (−0.03 to 0.17)	−0.04 (−0.14 to 0.05)
**Dynamic Tailoring**							
	On	87.0 (83.8 to 90.3)	37.7 (33.3 to 42.1)	16.6 (13.1 to 20.1)	58.7 (54.1 to 63.1)	18.6 (15.0 to 22.3)	36.6 (32.1 to 41.0)
	Off	83.1 (79.7 to 86.7)	36.8 (32.5 to 41.2)	10.3 (7.3 to 13.3)	52.1 (47.4 to 56.7)	13.2 (9.9 to 16.4)	30.9 (26.6 to 35.2)
	Raw difference	3.9 (−0.9 to 8.6)	0.9 (−53 to 7.1)	6.3 (1.7 to 10.9)^c^	6.6 (0.1 to 13.1)^d^	5.4 (0.5 to 10.3)^d^	5.7 (−0.5 to 11.9)
	SMD	0.08 (−.02 to 0.17)	0.01 (−0.08 to 0.10)	0.13 (0.04 to 0.23)^c^	.09 (0.00 to 0.19)^d^	.11 (0.01 to 0.20)^d^	.09 (−.01 to 0.18)
**Intensity**							
	Tapered	83.4 (79.9 to 86.9)	38.2 (33.7 to 42.6)	14.6 (11.2 to 17.9)	56.4 (51.8 to 60.9)	18.2 (14.6 to 21.8)	31.4 (27.1 to 35.7)
	Abrupt	86.8 (83.6 to 90.1)	36.4 (32.0 to 40.7)	12.3 (9.1 to 15.5)	54.3 (49.7 to 58.9)	13.6 (10.3 to 16.9)	36.1 (31.7 to 40.6)
	Raw difference	−3.4 (−8.2 to 1.4)	1.8 (−4.4 to 8.0)	2.3 (−2.3 to 6.8)	2.1 (−4.4 to 8.5)	4.5 (−0.4 to 9.5)	−4.8 (−10.9 to 1.4)
	SMD	−0.07 (−0.16 to 0.03)	0.03 (−0.06 to 0.12)	0.05 (−0.05 to 0.14)	0.03 (−0.06 to 0.12)	0.09 (−0.01 to 0.18)	−0.07 (−0.16 to 0.02)

^a^SMD: standardized mean difference.

^b^*P*<.001.

^c^*P*<.01.

^d^*P*<.05.

**Table 2:** Marginal effects of experimental design factors on key engagement metrics (95% CI).

Factor		Key Engagement Metric			
		Feature utilization, mean	Page views ≥25, %	Time on site ≥15 min, %	Returned to website, %
**Personalization**					
	On	2.38 (2.24 to 2.53)	31.6 (27.2 to 36.0)	31.3 (27.0 to 35.6)	37.9 (33.3 to 42.4)
	Off	2.43 (2.28 to 2.58)	32.9 (28.5 to 37.3)	28.4 (24.1 to 32.6)	39.1 (34.6 to 43.7)
	Raw difference	−0.05 (−0.25 to 0.16)	−1.3 (−7.5 to 4.9)	2.9 (−3.2 to 9.0)	−1.2 (−7.7 to 5.2)
	Standard mean difference	−0.021 (−0.115 to 0.074)	−0.017 (−0.111 to 0.077)	0.045 (−0.049 to 0.139)	−0.019 (−0.113 to 0.075)
**Integration**					
	On	2.59 (2.44 to 2.74)	30.8 (26.5 to 35.2)	29.7 (25.4 to 34.0)	37.0 (32.4 to 41.5)
	Off	2.22 (2.08 to 2.37)	33.7 (29.3 to 38.1)	29.9 (25.6 to 34.0)	40.0 (35.5 to 44.6)
	Raw difference	0.36 (0.16 to 0.57)^a^	−2.9 (−9.1 to 3.3)	−0.2 (−6.3 to 5.9)	−3.0 (−9.5 to 3.4)
	Standard mean difference	0.166 (0.071 to 0.260)^a^	−0.043 (−0.137 to 0.051)	−0.003 (−0.097 to 0.091)	−0.044 (−0.138 to 0.048)
**Dynamic Tailoring**					
	On	2.55 (2.40 to 2.70)	35.9 (31.4 to 40.4)	32.3 (28.0 to 36.7)	41.6 (36.9 to 46.2)
	Off	2.26 (2.12 to 2.40)	28.6 (24.3 to 32.9)	27.3 (23.1 to 31.0)	35.4 (30.9 to 39.9)
	Raw difference	0.29 (0.09 to 0.50)^b^	7.3 (1.1 to 13.5)^c^	5.0 (−1.0 to 11.1)	6.2 (−0.3 to 12.6)
	Standard mean difference	0.134 (0.040 to 0.229)^b^	0.110 (0.016 to 0.205)^c^	0.078 (−0.016 to 0.173)	0.089 (−0.004 to 0.184)
**Intensity**					
	Tapered	2.42 (2.27 to 2.57)	33.1 (28.72 to 37.6)	31.3 (27.1 to 35.7)	40.0 (35.5 to 44.6)
	Abrupt	2.39 (2.25 to 2.54)	31.4 (27.0 to 35.8)	28.3 (24.1 to 32.6)	36.9 (32.5 to 41.5)
	Raw difference	0.03 (−0.18 to 0.23)	1.7 (−4.5 to 8.0)	3.0 (−3.0 to 9.1)	3.1 (−3.3 to 9.5)
	Standard mean difference	0.012 (−0.083 to 0.106)	0.028 (−0.066 to 0.122)	0.047 (−0.047 to 0.141)	0.045 (−0.049 to 0.139)

^a^SMD: standardized mean difference.

^b^*P*<.001.

^c^*P*<.01.

The main effects of *intensity* and *personalization* failed to attain even nominal levels of statistical significance on any engagement metrics. However, the impact of these 2 experimental factors was still beneficial as a whole via their synergistic interactions with *dynamic tailoring* and *integration*. Examination of the factorial models for each interactive feature in isolation revealed a synergistic interaction between *integration* and *personalization* on Set Quit Date (*P*<.001) and a synergistic interaction between *dynamic tailoring*, *integration*, and *personalization* on Track Smoking Triggers (*P*=.02). A synergistic interaction of *dynamic tailoring* × *integration* × *intensity* × *personalization* was also detected for time on site (*P*=.01), while a synergistic interaction of *integration* and *personalization* was detected for time on return visits to the website (*P*=.04).

To better understand the joint effect of all 4 experimental factors, Table 3 (interactive features) and Table 4 (key engagement metrics) present point estimates and 95% CIs for arm-specific means based on simulation findings also depicted in Multimedia Appendices 1 and 2. As seen in Table 3, Arm 16 yielded the highest engagement rates for Set Quit Date, Build Support System, Track Smoking Triggers, and Beat Smoking Triggers. It lagged behind other arms in terms of the proportions of users at the high engagement level for Choose Quit Smoking Aid and Visit Community. Overall, Arm 16 had the highest rate of interactive feature use (2.95 out of 6).

As seen in Table 4, Arm 16 also had the greatest proportion of participants at the high engagement level for page views (45%) and return visits (51%), although it ranked second with regard to the proportion at the high engagement level for time on site (41%). Sensitivity analyses that varied the cutoffs for page views and time spent on site from the 55th to the 75th percentile (ie, from 15 to 32 pages and 8 to 20 min, respectively) confirmed the superiority of Arm 16, suggesting that these findings are robust to the choice of cutoff.

**Table 3:** Arm-specific interactive feature utilization rates (95% CI).

Arm	Factor^a^				Interactive Feature					
	P^b^	IG^c^	T^d^	IS^e^	Set quit date	Choose quit smoking aid	Build support system	Track smoking triggers	Beat smoking triggers	Visit community
1	−	−	−	−	95 (86-99)	21 (12-33)	3 (0-12)	48 (35-61)	14 (6-25)	41 (29-54)
2	−	−	−	**+**	88 (77-95)	43 (30-56)	6 (2-16)	48 (35-61)	6 (2-16)	32 (21-45)
3	−	−	**+**	−	86 (75-94)	28 (17-41)	8 (3-18)	55 (42-68)	12 (5-23)	39 (27-52)
4	−	−	**+**	**+**	90 (80-96)	30 (19-43)	12 (5-23)	61 (48-73)	21 (12-33)	45 (32-58)
5	−	**+**	−	−	85 (73-92)	54 (40-66)	10 (4-20)	57 (44-70)	8 (3-18)	34 (22-47)
6	−	**+**	−	**+**	75 (63-85)	45 (32-58)	7 (9-29)	65 (51-76)	21 (12-33)	23 (13-35)
7	−	**+**	**+**	−	81 (69-90)	43 (30-56)	23 (13-35)	52 (39-65)	17 (9-29)	37 (25-51)
8	−	**+**	**+**	**+**	85 (73-92)	52 (39-65)	19 (10-31)	59 (46, 71)	21 (12-33)	30 (19-43)
9	**+**	−	−	−	83 (71-91)	25 (15-37)	5 (1-14)	52 (39-65)	12 (5-23)	26 (16-39)
10	**+**	−	−	**+**	75 (63-85)	25 (15-37)	5 (1-14)	48 (35-61)	14 (6-25)	30 (19-43)
11	**+**	−	**+**	−	86 (75-94)	23 (13-35)	10 (4-20)	54 (40-66)	14 (6-25)	35 (24-49)
12	**+**	−	**+**	**+**	81 (69-90)	30 (19-43)	12 (5-23)	54 (40-66)	21 (12-33)	34 (22-47)
13	**+**	**+**	−	−	86 (75-94)	46 (34-60)	21 (12-33)	52 (39-65)	14 (6-25)	34 (22-47)
14	**+**	**+**	−	**+**	77 (65-87)	37 (25-51)	15 (8-27)	46 (34-60)	17 (9-29)	28 (17-41)
15	**+**	**+**	**+**	−	92 (82-97)	52 (39-65)	19 (10-31)	65 (51-76)	19 (10-31)	43 (30-56)
16	**+**	**+**	**+**	**+**	95 (86-99)	45 (32-58)	30 (19-43)	70 (57-81)	25 (15-37)	30 (19-43)

^a^For P, IG, and T, + implies On and − implies Off. For IS, + implies Tapered and − implies Abrupt.

^b^P: Personalization.

^c^IG: Integration.

^d^T: Dynamic Tailoring.

^e^IS: Intensity.

**Table 4:** Arm-specific summaries of key engagement metrics (95% CI).

Arm	Factor^a^				Key Engagement Metric			
	P^b^	IG^c^	T^d^	IS^e^	Feature utilization (mean)	Page views ≥25 (percent)	Time on site ≥15 min (percent)	Returned to website (percent)
1	−	−	−	−	2.21 (1.84-2.64)	34 (23-48)	23 (13-36)	47 (34-60)
2	−	−	−	**+**	2.23 (1.86-2.66)	28 (18-42)	19 (10-32)	36 (24-49)
3	−	−	**+**	−	2.29 (1.91-2.72)	35 (23-49)	24 (14-37)	49 (36-62)
4	−	−	**+**	**+**	2.58 (2.18-3.04)	44 (31-58)	44 (31-58)	44 (31-58)
5	−	**+**	−	−	2.47 (2.08-2.92)	28 (18-42	30 (19-44)	26 (16-40)
6	−	**+**	−	**+**	2.45 (2.06-2.9)	28 (18-4)	26 (16-39)	37 (25-51)
7	−	**+**	**+**	−	2.53 (2.13-2.98)	33 (22-47)	29 (19-43)	37 (25-51)
8	−	**+**	**+**	**+**	2.66 (2.25-3.12)	29 (18-42)	29 (18-42)	35 (23-48)
9	**+**	−	−	−	2.01 (1.66-2.41)	26 (16-39)	26 (16-39)	30 (19-43)
10	**+**	−	−	**+**	1.95 (1.61-2.35)	31 (20-44)	33 (21-46)	37 (25-50)
11	**+**	−	**+**	−	2.21 (1.84-2.64)	32 (21-46)	34 (23-48)	32 (21-46)
12	**+**	−	**+**	**+**	2.31 (1.93-2.74)	37 (25-50)	35 (23-48)	44 (31-58)
13	**+**	**+**	−	−	2.53 (2.13-2.98)	29 (19-43)	37 (25-51)	33 (22-47)
14	**+**	**+**	−	**+**	2.21 (1.84-2.64)	22 (12-35)	22 (12-35)	35 (23-49)
15	**+**	**+**	**+**	−	2.90 (2.47-3.38)	30 (19-43)	21 (12-33)	39 (27-52)
16	**+**	**+**	**+**	**+**	2.95 (2.52-3.44)	45 (32-59)	41 (29-55)	51 (38-64)

^a^For P, IG, and T, + implies On and − implies Off. For IS, + implies Tapered and − implies Abrupt.

^b^P: Personalization.

^c^IG: Integration.

^d^T: Dynamic Tailoring.

^e^IS: Intensity.

**Figure 1:**
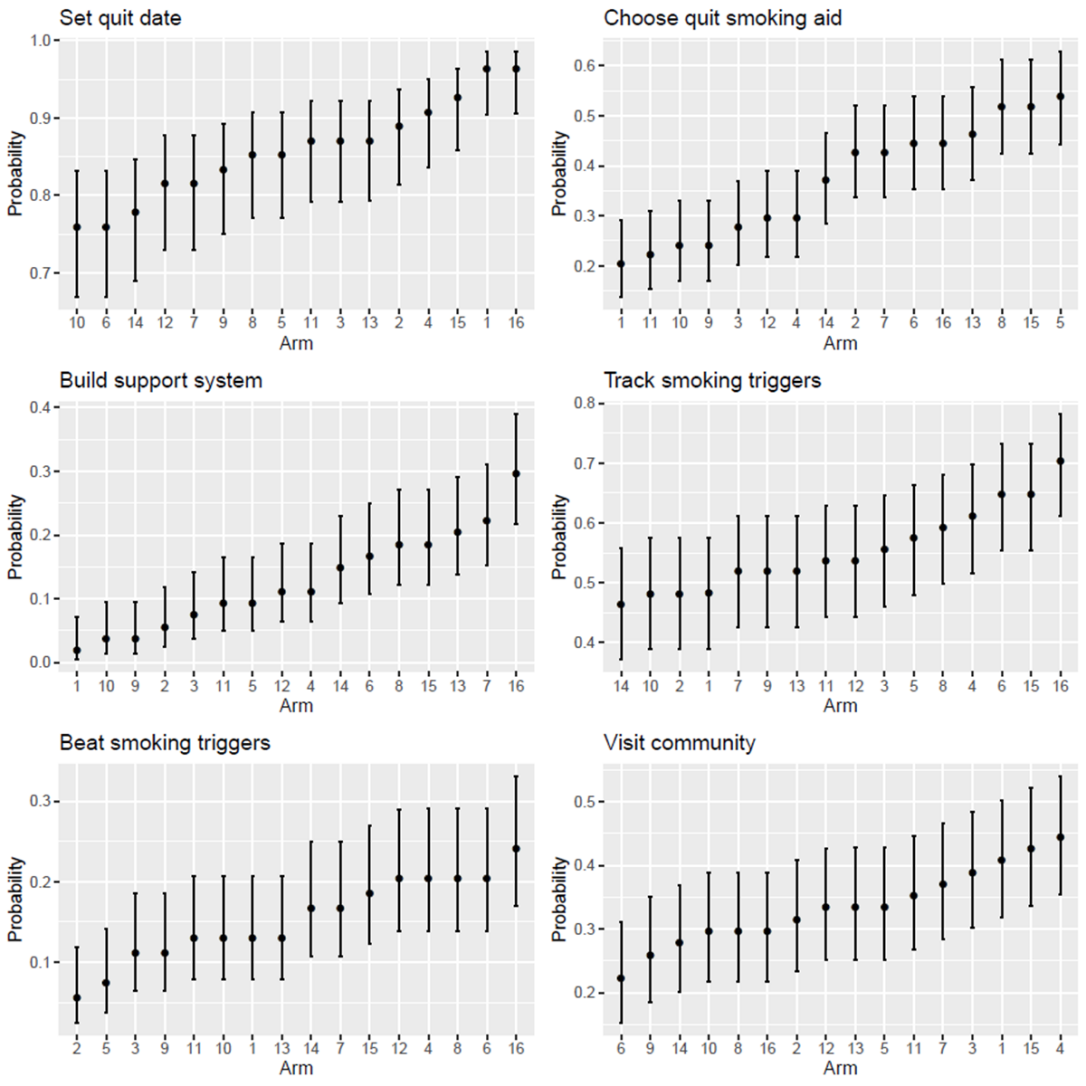
Arm-specific interactive feature utilization rates.

**Figure 2:**
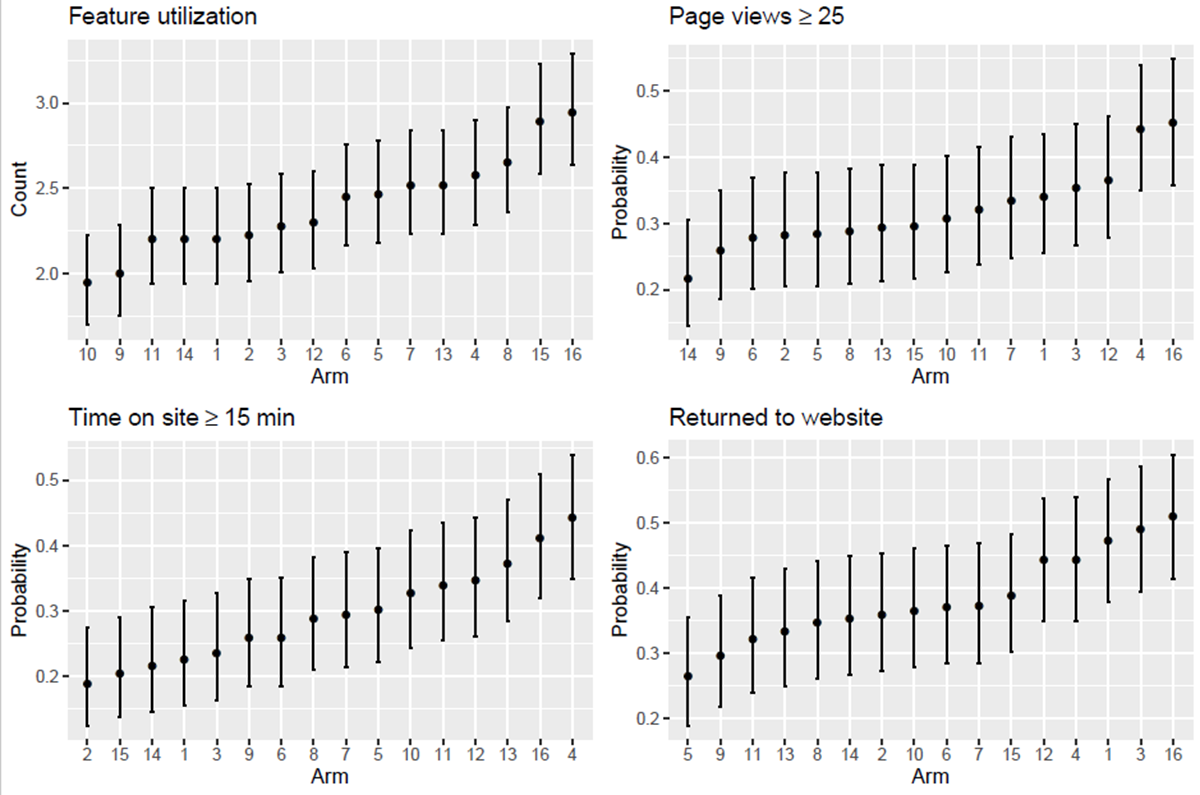
Arm-specific summaries of key engagement metrics (95% CIs).

Significance of pairwise differences at an alpha of .05 can be evaluated using the overlap method applied to the 83.5% CIs shown in Figures 1 and 2. The results confirmed the conclusion that Arm 16 was either the top-ranked arm or did not differ from the top-ranked arm for any of the key engagement metrics and individual interactive features across all 16 experimental conditions. However, this begs the question of whether the intervention as a whole led to a significant improvement in utilization outcomes over no intervention at all. To answer this question, we calculated Arm 1 versus Arm 16 SMDs for all outcomes of interest. We found that the combined effect of our 4 experimental factors on interactive utilization features was highest for Build Support System (SMD 0.56; 95% CI 0.27 to 0.81), followed by Choose Quit Smoking Aid (SMD 0.38; 95% CI 0.10 to 0.66) and Track Smoking Triggers (SMD 0.33; 95% CI 0.05 to 0.61). No significant effect was found for Beat Smoking Triggers (SMD 0.20; 95% CI −0.07 to 0.47), Set Quit Date (SMD 0.00; 95% CI −0.28 to 0.28), or Visit Community (SMD −0.17; 95% CI −0.44 to 0.10). As for our key engagement metrics, the largest effect was on overall feature utilization (SMD 0.33; 95% CI 0.06 to 0.59), followed by time on site (SMD 0.29; 95% CI 0.01 to 0.57), page views (SMD 0.16; 95% CI −0.11 to 0.44), and return visits to the website (SMD 0.05; 95% CI −0.22 to 0.33). As no SMD >.30 was observed for main factor effects on any of the outcomes of interest, these results also suggest that, for at least some outcomes, the combined intervention was stronger than individual factors alone.

## Discussion

### Principal Findings

The aim of this factorial screening experiment was to test the effectiveness of text message design factors in increasing treatment engagement among adult smokers who enrolled in an internet smoking cessation intervention. We examined general metrics of engagement (ie, page views, time on site, and return visits) and specific engagement metrics for core intervention components. As hypothesized, synergistic factor effects in Arm 16, in which all 4 experimental factors were active simultaneously, resulted in the highest rates of interactive feature use. Nearly all participants (95%) set a quit date, 70% tracked their triggers, and approximately half (45%) designated a medication plan. Arm 16 also yielded the greatest proportion of participants at high levels of engagement, with 40% to 50% of the sample engaged at the highest levels of page views, time on site, and return visits.

To our knowledge, this is the first study that has examined the impact of tailoring content based on treatment engagement. *Dynamic tailoring*, aimed at showcasing intervention features that participants had not yet used and encouraging ongoing utilization of those they had, was the most powerful of the 4 experimental factors tested in increasing engagement. It resulted in more participants engaging with the core components of tobacco dependence treatment, namely identifying and rallying the support of key people in their social network, identifying triggers for smoking, and developing coping strategies for those triggers. These results are consistent with previous research showing the effectiveness of individually tailored content and demonstrate that text messages can be an effective strategy to help shepherd and guide users through an intervention, much like would happen in a face-to-face encounter.

Although *dynamic tailoring* encouraged participants to explore the most program features, enabling users’ access to these features via text messages had the most dramatic impact on individual feature use. With a relatively simple mechanism to engage users in an interactive fashion via text messaging, *integration* was effective at increasing the utilization of interactive features above the study-wide mean. It increased the use of tools related to medication selection and planning for social support by 19 and 12 percentage points, respectively. The fact that we did not observe an impact of integration on setting a quit date likely results from a ceiling effect, given that 85% of all participants set a quit date, often immediately after website registration.

The lack of significant main effects for *personalization* and *intensity* is worth noting. Messages designed to feel individually tailored by using a person’s name, but where the content is clearly generic, did not appear to enhance program utilization. These findings are consistent with previous research that has shown that the use of a person’s name alongside generic information that is not perceived as personally relevant may even have counterproductive effects [45]. The fact that we only detected the synergistic effects of *personalization* when implemented alongside *dynamic tailoring* and *integration* is consistent with this notion. *Intensity* in this study was operationalized as the schedule of message delivery, and it was hypothesized that messages sent at less frequent and changing intervals over the 12-week intervention period would be more impactful than messages sent at a fixed interval with an abrupt drop-off. This hypothesis was not supported. It is possible that differences between the tapered and abrupt arms yielded variations in the dose of text messages received (eg, number of days enrolled, number of messages received), which we intend to explore in secondary analyses.

Finally, it is also worth noting that none of the experimental factors we tested increased engagement with the online community. For technical reasons, this was the only interactive feature that required the user to first visit the website to subsequently engage with community content via an SMS text message (ie, TIPS keyword). It may be that a text message approach that did not require a website action may have yielded different findings. Alternatively, it may be that interest in and use of social support resources—whether online or offline—may be a more trait-like characteristic that is not subject to external manipulation [46]. Other research has also failed to increase the use of an online social network in an experimental design [47,48].

### Limitations

Several limitations should be considered. This study does not allow us to draw conclusions about the impact of an engagement strategy versus none on smoking outcomes [10]. This factorial screening experiment was conducted as the first phase of 2-phase trial. Whereas all 16 arms were compared in terms of their ability to increase engagement with a smoking cessation intervention, the next phase of this study involves a comparative effectiveness trial (currently underway), which will allow us to evaluate the impact of the presence versus absence of a comprehensive engagement strategy (ie, Arm 16) in increasing abstinence rates. In addition, we cannot disentangle the effect of engagement messages alone because they were delivered as part of a broader text message intervention. This was a deliberate design decision because text messages solely focused on promoting engagement without reference to a user’s progress in quitting would likely have been perceived as irrelevant. Finally, we are cognizant of the fact that more does not always equal better when it comes to digital engagement [49,50]. Our classification of high levels of engagement was based on empirical distributions, which may not necessarily correlate with clinically meaningful engagement (ie, capable of promoting abstinence). In previous research, McClure et al [51] found positive effects of prescriptive message tone, dictated content viewing order, and reminder emails in a factorial screening experiment focused on internet engagement, but none of these features enhanced cessation outcomes [52]. Phase 2 of this study will enable us to evaluate the impact of the level of engagement on smoking outcomes, to investigate the role of complex issues such as reverse causality and confounding factors on the causal pathway from engagement to outcomes, and to determine what constitutes clinically meaningful engagement.

Given the proliferation of mobile apps for smoking cessation, one may question our use of text messaging as an engagement strategy over push notifications via a mobile app. Several factors support our decision. Text messaging is a recommended cessation modality [53], whereas the evidence for smartphone apps is lacking [15]. A majority of apps that are downloaded are either never opened or used only once [54]. In addition, text messaging may feel less intrusive to users and be more widely accepted: 57% of app users uninstall/decline to install apps because of privacy concerns [55]. Finally, smartphone penetration lags behind high rates of cellphone ownership [18].

### Comparison With Prior Work

Program completion results compare favorably with a large study from SmokefreeTXT [56], a US-based text message program from the National Cancer Institute. Among 25,283 individuals who subscribed to SmokefreeTXT, 38.3% (n=9686) completed the entire 42-day program. In our trial, 53.5% (461/864) of all study participants completed the full 90-day text message program. In both programs, a sizable number of participants disengaged early in treatment. We included all participants randomized to treatment in our analyses, whereas Augustson et al [56] restricted analyses to those that fully initiated treatment (ie, set a quit date and received first full day of treatment). Understanding patterns of early opt out from text message interventions and identifying opportunities for improving program delivery remain important areas of inquiry.

This study addresses several gaps in the literature on digital interventions. Previous studies on improving engagement have largely focused on the use of email and telephone calls [10]. The use of text messaging as an engagement strategy is novel in this regard. In addition, previous studies have suffered from small sample sizes and lack of statistical significance [10] and provided few insights into the characteristics of digital intervention approaches that make them effective for promoting engagement [19]. This study was conducted as a full factorial, which provided a reasonably powered and efficient opportunity to test for the presence of both main factor effects and pairwise interactions. Other smoking cessation trials involving a full factorial design [57] have also found that higher order interactions can account for more variance than the marginal effects. When interactions are significant, factors should not be examined in isolation, but one should consider their joint effects (ie, the sum of their main effects and multiway interactions). Although the MOST framework has been primarily described as an efficient approach for evaluating the main effects of intervention components, it can be easily adapted to accommodate higher order terms as well. Our results and those of Cook et al [57] suggest that synergistic interactions may often be present in smoking cessation trials and should be taken into account at the study design phase.

### Conclusions

In conclusion, this factorial screening experiment demonstrates the effectiveness of a theory-driven text message intervention in boosting overall engagement and use of the core features of an internet smoking cessation program among adult smokers. The results suggest that enabling users to engage with the tools and content of an internet intervention via text messages and tailoring the experience based on a user’s pattern of program use can boost the overall levels of engagement. These findings have relevance to improving engagement in internet health behavior change interventions more broadly and for future research into the complex relationship between engagement and outcomes. This study can serve as a model for conducting rigorous, fully powered research on engagement as a first step in understanding how to optimize behavior change outcomes in digital interventions.

## Appendix

Multimedia Appendix 1 Simulation-Based Histograms of Arms-Specific Utilization Rates: Interactive Features.

Multimedia Appendix 2 Simulation-Based Histograms of Arms-Specific Utilization Rates: Key Engagement Metrics.

## Abbreviations

EX: BecomeAnEX
MOST: Multiphase Optimization Strategy
SMD: standardized mean difference
