# Correction: Optimizing Text Messages to Promote Engagement With Internet Smoking Cessation Treatment: Results From a Factorial Screening Experiment

**DOI:** 10.2196/21027

**Published:** 2020-07-28

**Authors:** Amanda L Graham, George D Papandonatos, Megan A Jacobs, Michael S Amato, Sarah Cha, Amy M Cohn, Lorien C Abroms, Robyn Whittaker

**Affiliations:** 1 Innovations Center Truth Initiative Washington, DC United States; 2 Mayo Clinic College of Medicine and Science Rochester, MN United States; 3 Center for Statistical Sciences Brown University Providence, RI United States; 4 Oklahoma Tobacco Research Center University of Oklahoma Health Sciences Center Oklahoma City, OK United States; 5 Department of Prevention and Community Health Milken Institute School of Public Health The George Washington University Washington, DC United States; 6 National Institute for Health Innovation University of Auckland Auckland New Zealand

The authors of “Optimizing Text Messages to Promote Engagement With Internet Smoking Cessation Treatment: Results From a Factorial Screening Experiment” (J Med Internet Res 2020;22(4):e17734) noticed several errors in their published manuscript which had been introduced after proofreading. The following corrections have been implemented:

The symbols μ and φ were presented as m and f, respectively, in the following sentence of the Methods section; additionally, in this section, the negative sign was incorrectly subscripted:

SMDs for frequency counts were calculated as (*m*_1−_*m*_2_)/[*f*(*m*_1_+*m*_2_)]^1/2^, where *m*_1_ and *m*_2_ were the sample means of each comparison group.

This has been revised to:

SMDs for frequency counts were calculated as (μ_1_−μ_2_)/[φ(μ_1_+μ_2_)]^1/2^, where μ_1_ and μ_2_ were the sample means of each comparison group.

In the same paragraph, the negative sign was again incorrectly subscripted in the following sentence:

SMDs for binary outcomes were calculated as (p_1−_p_2_)/[p_1_×q_1_+p_2_×q_2_]^1/2^, where p_1_=1−q_1_ and p_2_=1−q_2_ were the sample outcome prevalence of each comparison group.

This has been revised to:

SMDs for binary outcomes were calculated as (p_1_−p_2_)/[p_1_×q_1_+p_2_×q_2_]^1/2^, where p_1_=1−q_1_ and p_2_=1−q_2_ were the sample outcome prevalence of each comparison group.

Additionally, due to a technical error the following sentence was published in the Results section of the Abstract:

As no SMD &gt;0.30 was observed for main effects on any outcome, results suggest that for some outcomes, the combined intervention was stronger than individual factors alone.

This has been revised to:

As no SMD >0.30 was observed for main effects on any outcome, results suggest that for some outcomes, the combined intervention was stronger than individual factors alone.

The correction will appear in the online version of the paper on the JMIR website on July 28, together with the publication of this correction notice. Because this was made after submission to PubMed, PubMed Central, and other full-text repositories, the corrected article has also been resubmitted to those repositories.

