# Supplementary material for: Optimizing Text Messages to Promote Engagement With Internet Smoking Cessation Treatment: Results From a Factorial Screening Experiment
Source: J Med Internet Res. 2020 Apr 2;22(4):e17734. doi: 10.2196/17734 (PMC7386536; doi:10.2196/17734)
Supplement: Multimedia Appendix 1 [file jmir_v22i4e17734_app1.pdf]

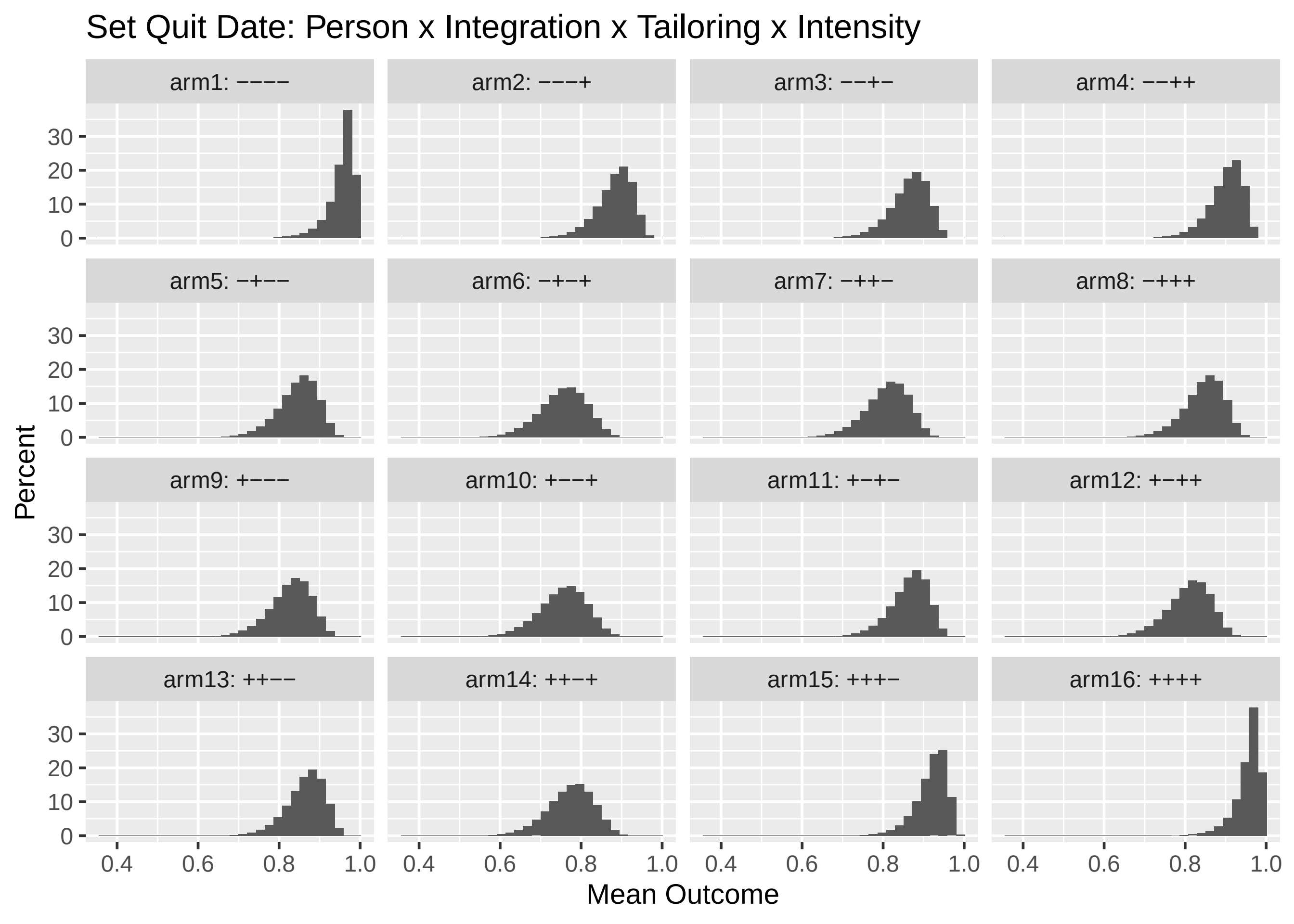

Set Quit Date: Person x Integration x Tailoring x Intensity
arm1: −−−−
arm2: −−−+
arm3: −−+−
arm4: −−++
arm5: −+−−
arm6: −+−+
arm7: −++−
arm8: −+++
arm9: +−−−
arm10: +−−+
arm11: +−+−
arm12: +−++
arm13: ++−−
arm14: ++−+
arm15: +++−
arm16: ++++
Percent
0
10
20
30
0.4
0.6
0.8
1.0
Mean Outcome

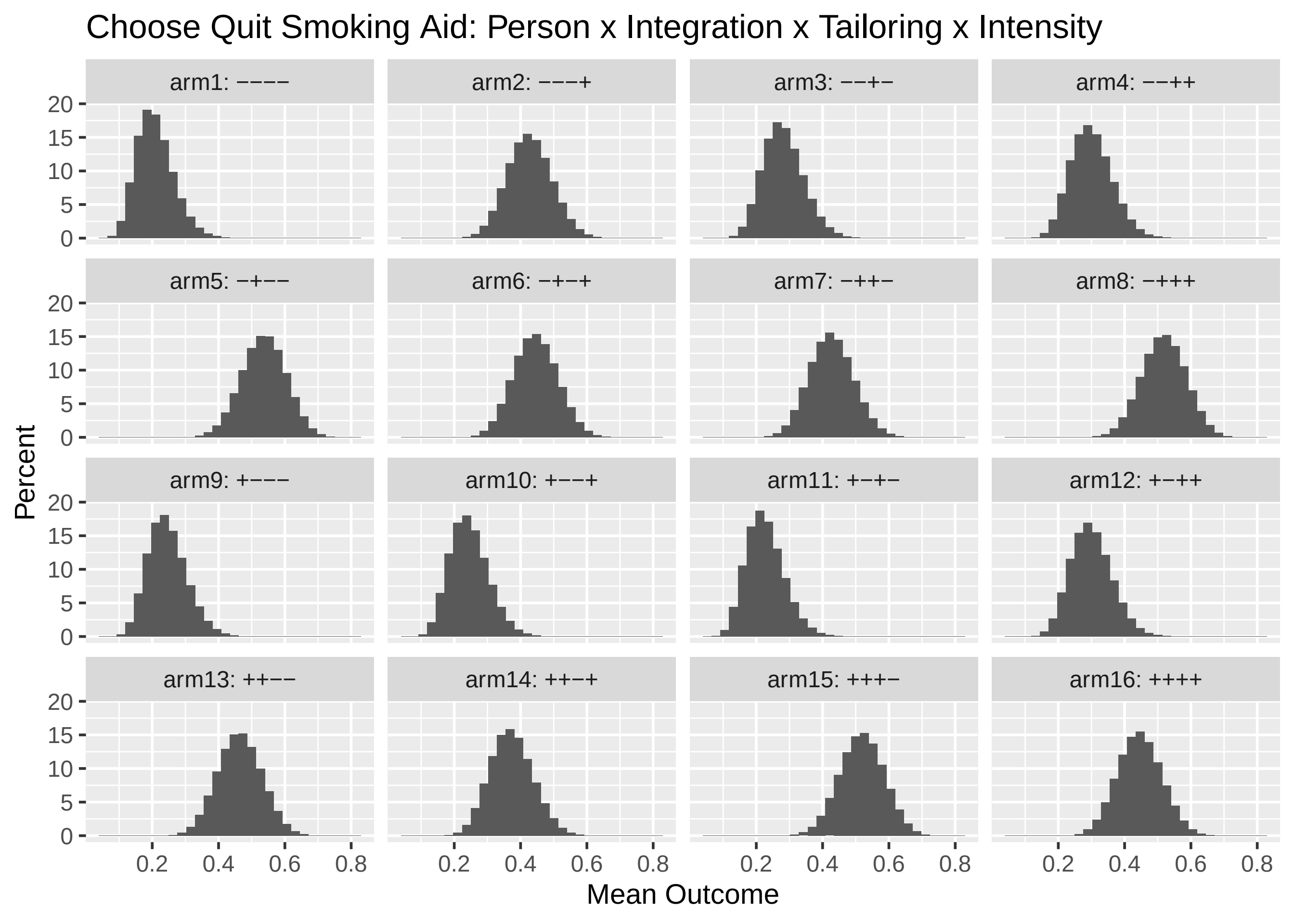

Choose Quit Smoking Aid: Person x Integration x Tailoring x Intensity
arm1: −−−−
arm2: −−−+
arm3: −−+−
arm4: −−++
arm5: −+−−
arm6: −+−+
arm7: −++−
arm8: −+++
arm9: +−−−
arm10: +−−+
arm11: +−+−
arm12: +−++
arm13: ++−−
arm14: ++−+
arm15: +++−
arm16: ++++
Percent
0
5
10
15
20
0.2
0.4
0.6
0.8
Mean Outcome

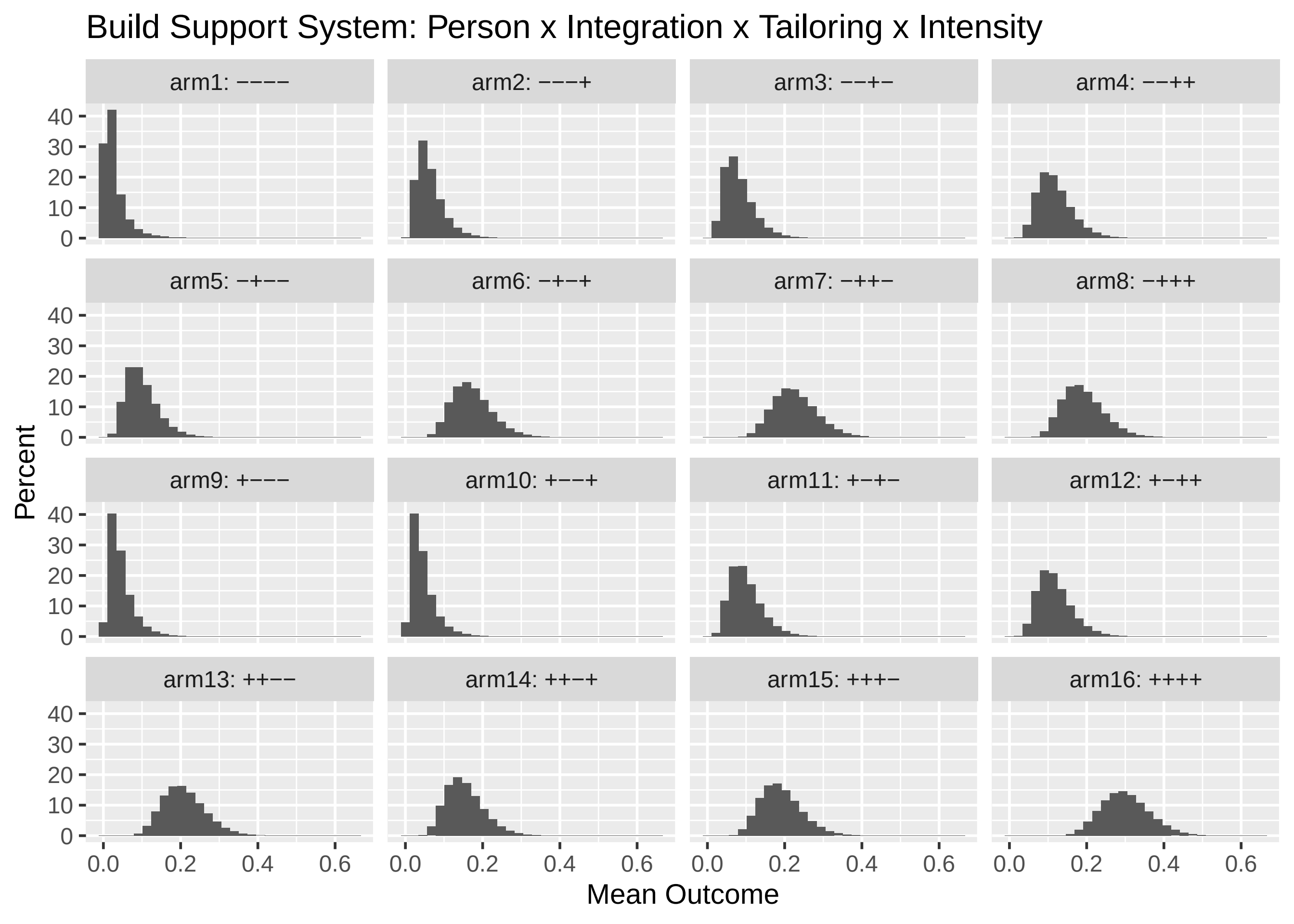

Build Support System: Person x Integration x Tailoring x Intensity
arm1: −−−−
arm2: −−−+
arm3: −−+−
arm4: −−++
arm5: −+−−
arm6: −+−+
arm7: −++−
arm8: −+++
arm9: +−−−
arm10: +−−+
arm11: +−+−
arm12: +−++
arm13: ++−−
arm14: ++−+
arm15: +++−
arm16: ++++
Percent
0
10
20
30
40
0.0
0.2
0.4
0.6
Mean Outcome

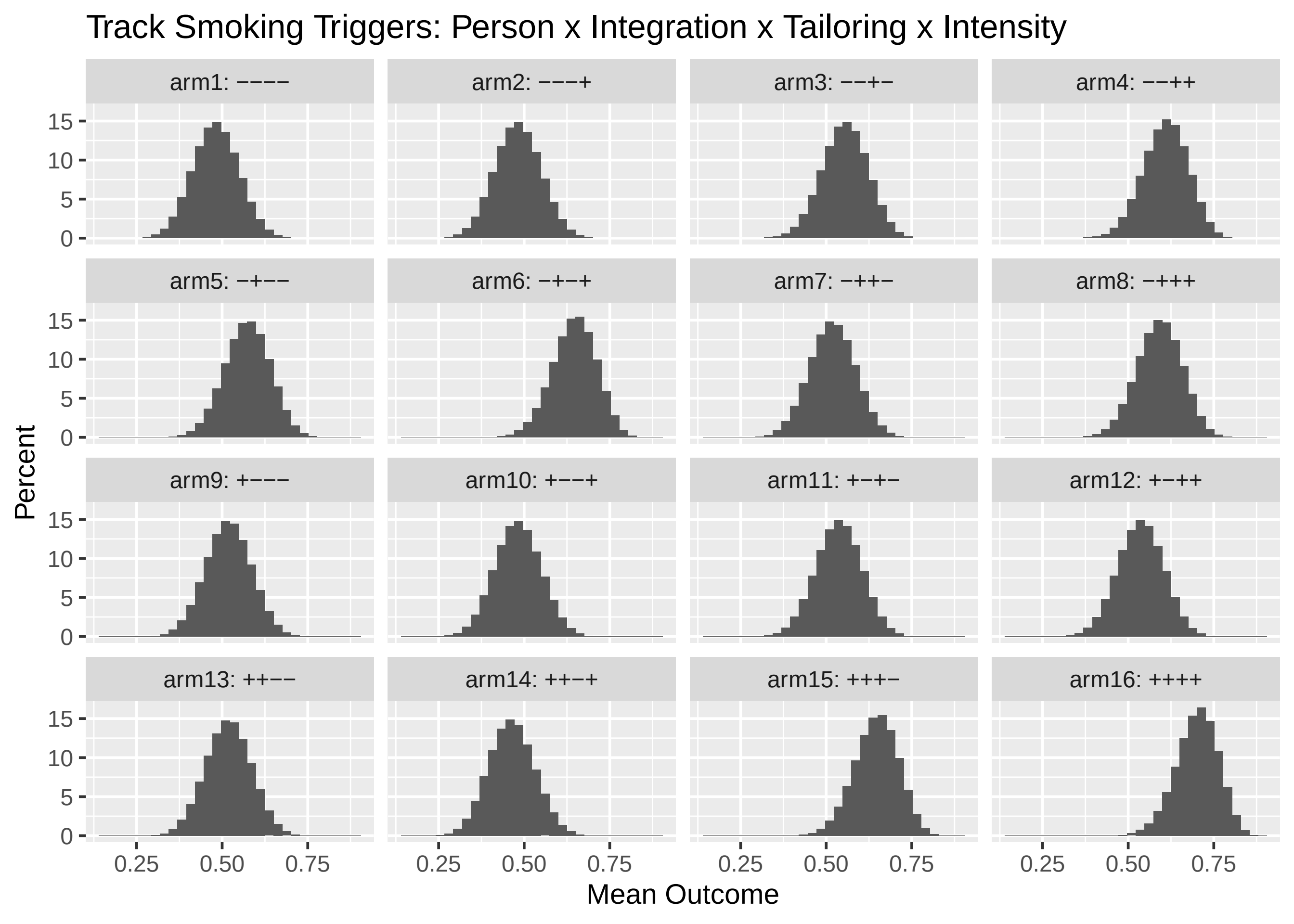
Track Smoking Triggers: Person x Integration x Tailoring x Intensity
arm1: −−−−
arm2: −−−+
arm3: −−+−
arm4: −−++
arm5: −+−−
arm6: −+−+
arm7: −++−
arm8: −+++
arm9: +−−−
arm10: +−−+
arm11: +−+−
arm12: +−++
arm13: ++−−
arm14: ++−+
arm15: +++−
arm16: ++++
Percent
0
5
10
15
0.25
0.50
0.75
Mean Outcome

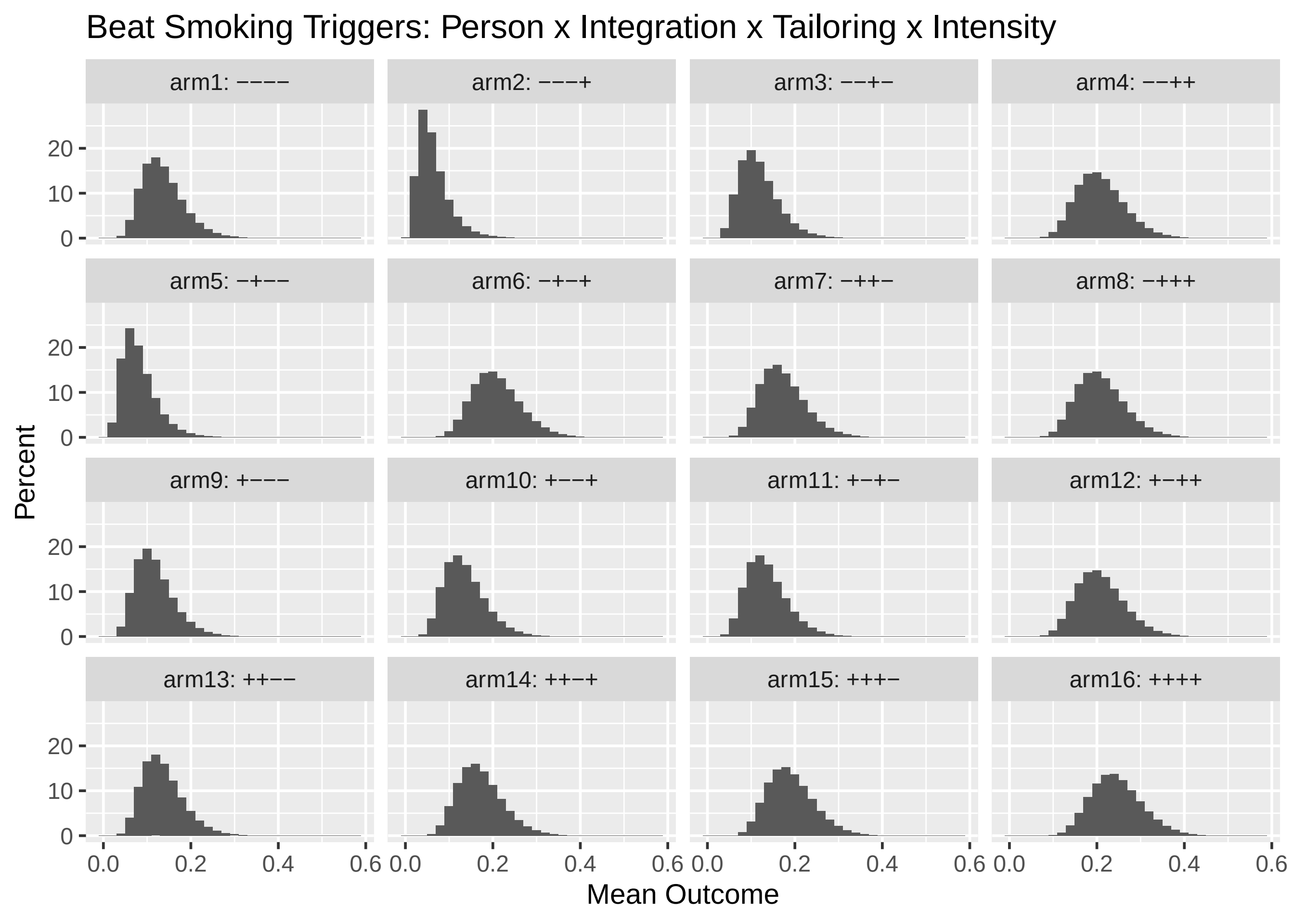

Beat Smoking Triggers: Person x Integration x Tailoring x Intensity
arm1: −−−−
arm2: −−−+
arm3: −−+−
arm4: −−++
arm5: −+−−
arm6: −+−+
arm7: −++−
arm8: −+++
arm9: +−−−
arm10: +−−+
arm11: +−+−
arm12: +−++
arm13: ++−−
arm14: ++−+
arm15: +++−
arm16: ++++
Percent
0
10
20
0.0
0.2
0.4
0.6
Mean Outcome

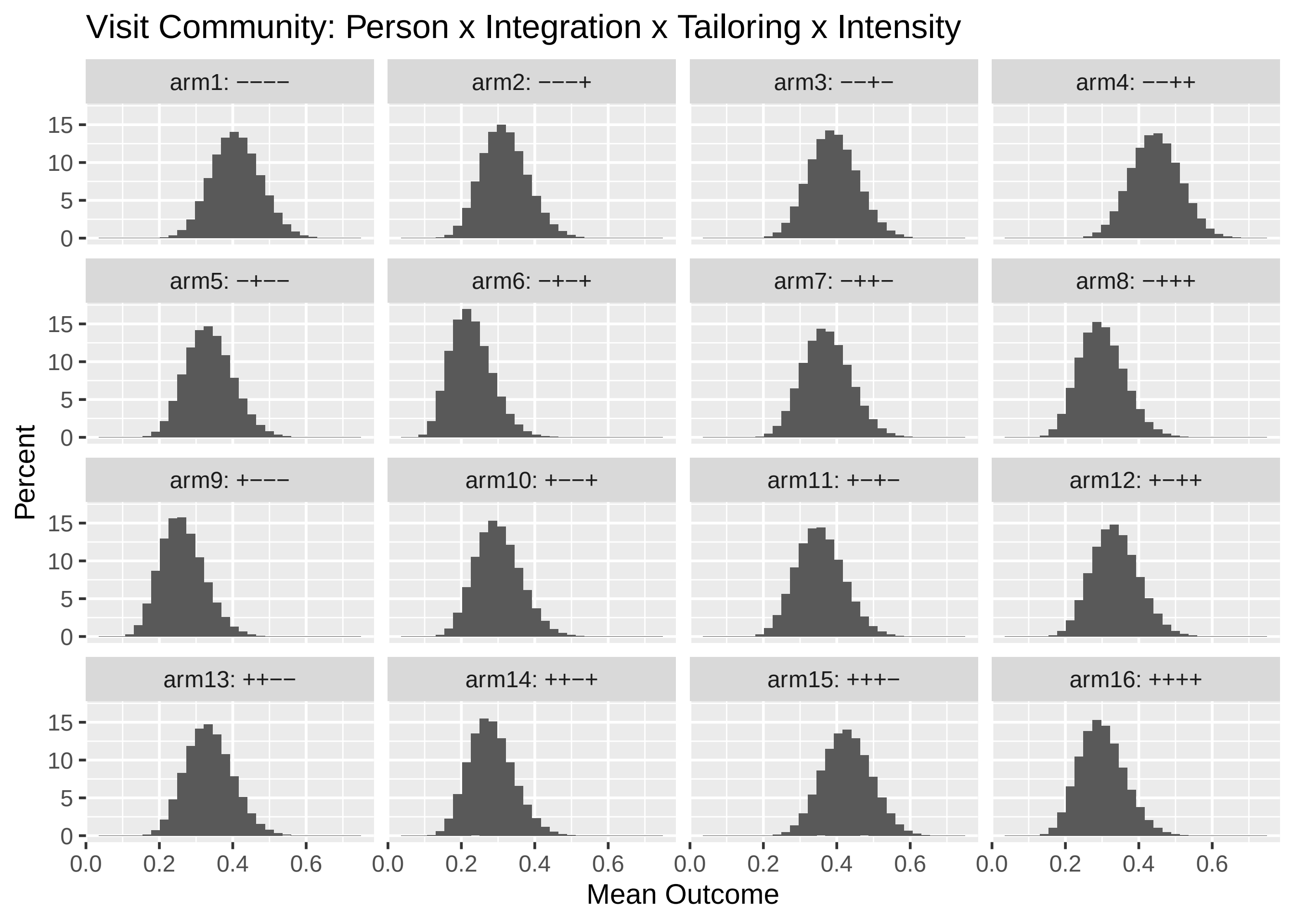
Visit Community: Person x Integration x Tailoring x Intensity
arm1: −−−−
arm2: −−−+
arm3: −−+−
arm4: −−++
arm5: −+−−
arm6: −+−+
arm7: −++−
arm8: −+++
arm9: +−−−
arm10: +−−+
arm11: +−+−
arm12: +−++
arm13: ++−−
arm14: ++−+
arm15: +++−
arm16: ++++
Percent
Mean Outcome
0
5
10
15
0.0
0.2
0.4
0.6
